# Steps on the Critical Path: Arresting HIV/AIDS in Developing Countries

**DOI:** 10.1371/journal.pmed.0010010

**Published:** 2004-10-19

**Authors:** Julie Gerberding

## Abstract

The director of the United States Centers for Disease Control and Prevention gives a personal view of how the world should tackle the HIV pandemic

As an intern, I took care of the first patients with HIV/AIDS at San Francisco General Hospital, and so I grew up with AIDS in the early days of my medical career. We struggled through the confusion about what was making people so sick, and each new day brought a new discovery about the disease and its consequences. I went through that evolutionary process along with everybody else, and it shaped me in many profound ways. Before long, I recognized that this wasn't a disease of “those people over there.” This was a disease that could strike anyone, anytime. And as physicians, we had to adjust our thinking about our own vulnerability to occupational risk, and to emphasize prevention, because there wasn't going to be a cure for a long, long while. And not only physicians had to rethink things—AIDS has reshaped society's very notions of the most basic human behaviors.

I was in Uganda in 1985, early in the AIDS epidemic there. We knew then where that epidemic was going to go, absent an effective vaccine or cure, but few of us could have imagined that it would evolve so quickly without an end in sight. While the people of Africa have achieved a huge amount in tackling HIV/AIDS, particularly in Uganda, the epidemic is far from being under control on that continent and is spreading through other parts of the world with alarming speed.

## The Crisis of Human Resources

The theme of this year's International AIDS Conference in Bangkok was “Access for All.” Over the past few years, it has become increasingly apparent that a critical component of assuring access to care and treatment is human capital. Like fiscal capital, human resources are essential to ending the AIDS pandemic. I visited Africa with US Health and Human Services Secretary Tommy Thompson and many AIDS experts last December, and we saw evidence of this critical need in every country we visited. The miracles of modern science are meaningless without systems and people to deliver them to those in need.[Fig pmed-0010010-g001]

**Figure 1 pmed-0010010-g001:**
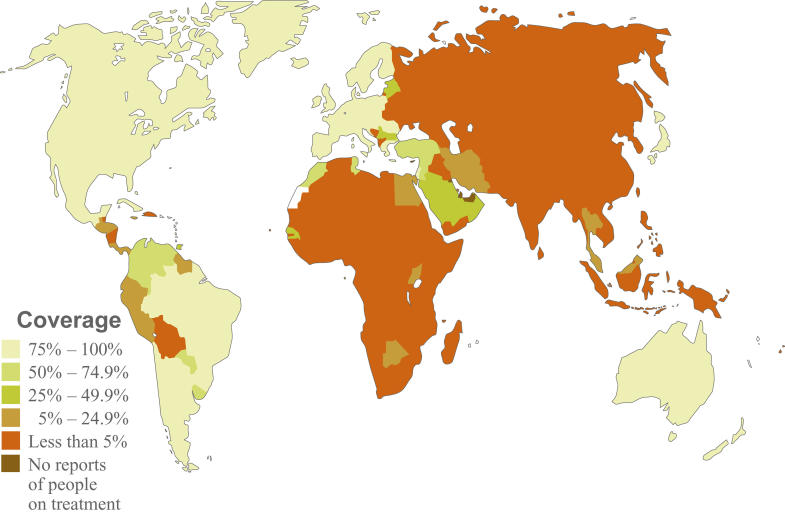
Estimated Percentage of Adults in Need of Antiretroviral Treatment Who Are Receiving It, as of March of 2004 (This graphic is based on an image by the World Health Organization, available at http://www.who.int/3by5/en/coverage_march2004.jpg)

The World Health Organization estimates that of the 40 million people worldwide infected with HIV, 6 million need immediate, life-sustaining antiretroviral therapy. Fewer than 400,000 people in developing countries have access to such treatment (Figure 1) [1,2]. There are too few skilled health care workers to provide reliable delivery and administration of these life-saving therapies. According to a recent Institute of Medicine report, and a study sponsored by the US Agency for International Development, the number of health care workers in many African countries is actually shrinking as they are lured to developed countries by better pay and professional opportunities (Box 1) [2,3]. Reversing this brain drain is essential over the long-term, as HIV treatment and care will be required for decades. In the short-term, the Institute of Medicine called for expanded efforts “to bring qualified volunteer initiative medical professionals into both urban and rural areas to support prevention, care, and training programs” [2]. I could not agree more that addressing the human resource needs will be essential as we move forward—and not just for HIV/AIDS programs, but for all aspects of public health and health care.[Boxed-text box1]

**Box 1. The Brain Drain: Facts and Figures [2,3]**

- Only 360 of the 1,200 doctors trained in Zimbabwe during the 1990s continue to practice within the country.
- Two-thirds of University of Zimbabwe medical students intend to leave the country after graduating, and one of the country's major 1,000-bed teaching hospitals lacks a single qualified pharmacist.
- In Zambia, only 50 of the 600 doctors trained locally since independence have remained in the country.
- In Ghana, 320 nurses are recorded to have been lost in 1999, roughly equivalent to that country's annual output of nurses; losses for the year 2000 totaled 600.

It has now been shown, beyond any doubt, that even in resource-poor countries with the most basic health infrastructure, people get the same benefit from treatment and prevention interventions as those in the rich world [4]. In fact, surveys in Cape Town, Kampala, Khayelitsha, and Senegal found rates of adherence to antiretroviral therapy of 90%–94%, compared with estimates of 70% in developed countries [5,6,7].

## When You Have Seen the Faces

We hear the numbers—the millions upon millions infected—and we grow numb. That is why we must go to the front lines—the households and communities—and start focusing on each individual living with HIV. I was at the dedication of an AIDS clinic in Kenya. It was raining, and we were waiting outside with our umbrellas. A 12-year-old girl in front of me turned around and leaned her head against my belly and said, “Could you take me to America? I need drugs.” If you take that girl's face and multiply it a thousand times—that is the memory I bring home from Africa: the faces of the children and their asking, “Why are so many of our parents dying? Why are we dying?”

We visited a US Centers for Disease Control and Prevention (CDC) program in the very remote areas of Uganda where there are no roads and it is impossible for people to come into population centers to receive HIV testing and other services. Young staff from the CDC are working with Ugandans and community organizations in that area to deliver antiretroviral therapy. Some may think that the difficulties of delivering antiretroviral therapy into such a remote area are overwhelming—and some may question whether this is a sustainable intervention. But once you see firsthand what miracles are possible, your world view changes almost overnight.

What we saw was the success of a wonderful home-based treatment and care program for people who don't have access through other means. And when I say “home-based care,” picture a hut without running water or electricity, where only motorcycles are available to deliver medications. The first step of the program is to provide clean water. Coliforms and other pathogens in the water supply for the household are removed through an inexpensive water vessel fitted with a filter and through a chlorination process. In addition, a cotrimoxazole tablet is given every day, which, in one patient's words, changed his life because he began to feel well almost immediately. Not only do the cotrimoxazole prophylaxis and the water treatment improve diarrheal illness, but malarial parasitemia also drops. So that is a very positive, unexpected consequence of just two very simple and inexpensive interventions. Many patients with HIV/AIDS in Africa also have tuberculosis and are put on tuberculosis therapy in addition to cotrimoxazole. As a result, they begin to feel better even before they begin antiretroviral therapy.[Fig pmed-0010010-g002]

**Figure pmed-0010010-g002:**
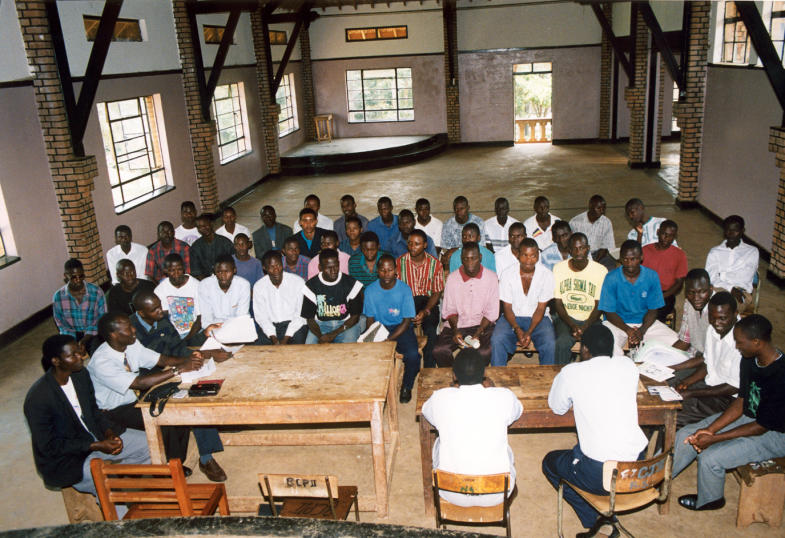
Behavior change club at a technical school in Entebbe, Uganda (Photo: Arjen van de Merwe/World Population Foundation)

We spent time with one of the patients in the home-based care program. As she began to participate in these programs, tests became available to measure her CD4 count. She explained to me what her CD4 count was, what it meant, and how it improved when she started the cotrimoxazole and tuberculosis therapy. She had begun taking three antiretroviral drugs and held up her pill box to explain her regimen in detail. Every week a Ugandan health aide delivered her supply of pills on a CDC motorcycle and monitored her adherence to the treatment. Not only was she extremely reliable in taking her medications, but she also knew more about them and their side effects than most of the patients I treated at San Francisco General. She was also an expert in HIV prevention. I asked her, “What do you do to protect your three young sons from this infection?” She replied, “Every day I take them by the hand, and I go out of the house and I say, ‘Do you see that mound of dirt? That is your father’s grave. Your father acquired this fatal infection through sex. Be careful.'” And then she talks to them about the “ABCs” (“A” for abstinence, “B” for being faithful, “C” for condoms).

So when you see a story like that unfold in the middle of Africa, it's impossible not to be hopeful. And yet, it's also very sobering because we are reminded of our responsibility. The question is not what the international health community is accomplishing in these countries now, but what we could accomplish if we joined together to really fight this war on AIDS. Such a story also inspires hope because you can see the multiplier effect that comes from taking on one problem and can see the way that effort can expand to encompass and address a much greater set of problems.

## Beyond ABCs—Diagnosis and Responsibility

When we think about successful prevention models in Uganda, “ABC” certainly stands out [8]. However, at this point in the epidemic curve, other letters must also be considered. Most HIV transmission is accounted for by infected people having risky sex with uninfected people. Both in the US and in Africa, studies show that most infected people engaging in risky sex are unaware of their infection status, and that when their infection is diagnosed, they usually take steps to protect the others with whom they are having contact [9,10,11,12]. So let's add the letter “D” for diagnosis. In fact, improving efforts to help people choose risk avoidance and to diagnose those who are already infected is the cornerstone of the CDC's new domestic HIV prevention strategy. Diagnosis is extremely important in many African communities, especially where the number of discordant couples—where one individual is infected and the other is not—is high. Sadly, many couples “being faithful” now do not realize that one partner is already infected and are not being reached with diagnostic testing programs. So “ABCD” is a concept that I would like to put out on the table as food for thought. Of course, there is another letter that we need to stress: the letter “R,” for responsibility: personal sexual responsibility is a critical component of HIV prevention. Many women and girls become infected after being raped by men or because their social circumstances rob them of the power to refuse sex. Men must be held accountable for greater sexual responsibility and for ending sexual violence and degradation of women and girls. HIV prevention programs need to emphasize responsibility, but not lose sight of the fact that responsibility can be practiced only with personal autonomy, which many women and girls simply do not have.

## Expanding the Team to Meet the Needs

The innovative programs and ideas emerging in Africa can change the picture of the AIDS epidemic. The purchase of antiretroviral drugs for Africans is not the big challenge. Access to drugs will improve in Africa. The real challenges are delivering drugs in a safe and effective way, monitoring therapy, and sustaining the pipeline of drugs so that ongoing treatment can be guaranteed. In the example of the home-based program in Uganda, we have seen that these challenges can be overcome. Expanding access to prevention, care, and treatment services isn't going to be easy, but it is certainly possible. It will take unprecedented commitment by people in the public sector, the private sector, faith communities, and community organizations, and perhaps most importantly, individual volunteers who make up their minds to contribute in any way they can.

Last fall, the US Peace Corps announced that it was activating programs in some countries that allow volunteers to help communities fight the AIDS epidemic, but this is just one of many steps that are being taken. The US president's Emergency Plan for AIDS Relief will provide $15 billion, including almost $10 billion in new funds, over five years for international AIDS assistance [13], and I am part of the team that is charged with making this plan happen. I look forward to learning from others in the global health community how we can best expand our impact and collectively find a way to support the delivery of prevention messages and life-saving medications to everyone in Africa—and especially to that little girl at the Kenyan clinic who touched my heart.

## Abbreviations

CDC: Centers for Disease Control and Prevention
